# Supplementary material for: Differences in perceptions and acceptance of COVID-19 vaccination between vaccine hesitant and non-hesitant persons
Source: PLoS One. 2023 Sep 8;18(9):e0290540. doi: 10.1371/journal.pone.0290540 (PMC10490972; doi:10.1371/journal.pone.0290540)
Supplement: S1 Appendix — (DOCX) [file pone.0290540.s001.docx]

Appendix I

Focus Group Guide

**Introduction:** Hello my name is {moderator’s name}. I do quality improvement projects at the University to help our medical center improve their services.

**Purpose:** Thank you so much for agreeing to talk with us about your beliefs and experiences around vaccinations, particularly the COVID-19 vaccine. The purpose of this project is to learn about individual’s thoughts and opinions about getting or not getting vaccinated against COVID-19. There are no right or wrong answers. We just want to hear your honest thoughts.

**Confidentiality**: The focus groups will take one hour. Your participation is completely voluntary. We will ask you about your experiences with COVID vaccinations. When we summarize the information in a report, we will not be using any names, and your answers will be combined with everyone else’s, so no one will know that you were included in this project. [For Veterans: Please know that the information you share with us today will have zero impact on your Veteran benefits.] Your participation is voluntary, confidential, and anonymous.

Do you have any questions before we begin?

**Consent to Interview:** Do you agree to be in this focus group?

**Consent to Record**: We would like to record this focus group so that we accurately capture the information you provide us. Are you okay with that?

[After obtaining verbal consent – **START RECORDING**]

I have started recording. Today’s date is [M/D/Y] and this is focus group [ID#]. I’d like to confirm; do I have your permission to record this focus group?

We would like to start by discussing your attitudes toward the vaccine. There are no rights or wrongs here and we value each person’s viewpoint. The goal is just to help us better understand a person’s decisions.

1. In what ways has COVID-19 impacted your day-to-day life?
2. How would you describe your likelihood of being exposed to COVID-19?
   - [Probe: How is this influenced by your work and/or living contexts?]
3. In general, what do you think about the safety and effectiveness of vaccines (such as those for seasonal influenza, HPV, measles, etc.)?
   - Do you typically get vaccines, like an annual flu shot? Why or why not?
   - Have you had any negative experiences with vaccines in the past?
     - If YES, what happened?
       - [Probe: Did you have an allergic reaction? Side effects? Did you receive unclear information or misinformation?]

[Following five questions were asked to educators instead of the first three questions]

1. How has your experience been working in the schools during the pandemic?

1. Do you feel your institution took hygiene and safety into sufficient consideration?

1. Were you asked or felt compelled to return and if so what was that experience like?

1. How do you feel your students (remote or in-person) have been handling education during the pandemic?

1. What would be the one thing you would want your administration to know about your teaching experience during the pandemic?
2. As of today, have you received one or both doses of the COVID-19 vaccine?
   - - [If eligible] Have you scheduled an appointment to get the COVID-19 vaccine?
     - [If only one dose] Why haven’t you received the second dose yet?
   - IF NO: What barriers might prevent you from getting vaccinated or receiving your second dose?
     - [Probe: Are there any logistical challenges such as transportation, time limitations, or work/familial obligations?]
     - [Probe: Do you have family members who might strongly discourage you from getting vaccinated?)
   - IF NO: Do you currently plan on getting vaccinated for COVID-19?
     - If NO, is this due to a current medical condition or personal vaccine history?
     - If NO, what factors, if any, might change your mind about getting it?
       - What would help you feel more motivated to get vaccinated in the near future? [Probe: What would be the tipping point for you to get vaccinated?]
       - Who could influence you to be more comfortable getting vaccinated?
     - If YES, Do you have a preference for receiving one of the vaccines?
3. Has your attitude towards getting vaccinated changed since you first learned about any of the vaccines?
   - If YES, Can you please tell me about how your thinking has changed over time?
     - [Probe: what factors led to this change?]
     - Were there specific people or conversations that changed your mind?
4. What personal risks, if any, do you associate with getting a COVID-19 vaccine?
   - [Probe: What do you know about the side effects?]
5. What benefits, if any, do you associate with the COVID-19 vaccine?
   - [Probe: Do you believe the vaccine will prevent you from contracting COVID-19?]
   - [Probe: Do you believe the vaccine will prevent severe illness or death if you get COVID-19?]
   - [Probe: What positive changes, if any, do you anticipate that getting vaccinated might have on your day-to-day life? In the short-term? Long-term?]
6. What thoughts do you have about the way the vaccine is being offered locally?
   - [Probe: How well do you think the state/VA has done in making it easy for individuals to get vaccinated?]
   - [Probe: What suggestions would you have to make the process better?]
7. What information have you have received regarding the vaccine in the past couple months?
   - [Probe: Consider the clarity, frequency, and content of this information]
   - [Probe: Was it more informational or encouraging vaccination? Or a combination of both?]
   - [Probe: What else would you like to have seen?]
   - [Probe: through news sources or social media channels such as Twitter or Facebook?
8. Which people or sources do you trust the most to give you accurate information about COVID-19 vaccination (e.g., its safety, whether it is a good idea for you to get it, etc.)?
   - Have you had conversations about the vaccine with family members or friends?
     - [e.g., spouse/partner, children, parents, family, friends, church/community group, or providers]
   - Have you spoken with a provider about the COVID-19 vaccine? If yes, what was that conversation like?
     - [Probe: Did you feel that your questions were answered, and your concerns were addressed?]
     - [Probe: Was it more informational or encouraging vaccination? Or a combination of both?]
     - [Probe: What type of provider did you speak to? (Primary care, nurse, specialist, mental health, etc.)]
   - Which ones do you not trust?
9. [For Veteran women groups: A lot of women have been concerned about how the vaccine impacts fertility. What do you feel about these claims?]
10. Do you have any other thoughts regarding the COVID-19 vaccine that you would like to share with me today?

*Thank you for participating in this focus group. I’m going to turn off the recording now.*

[For non-Veteran sample only] Share demographic form with participants:

How old are you?

Are you Hispanic or Latino?

How would you describe your race? [e.g., White, Black, American Indian, etc.]

How would you describe your gender?

What is the highest level of education you have completed?
