# Supplementary material for: Differences in perceptions and acceptance of COVID-19 vaccination between vaccine hesitant and non-hesitant persons
Source: PLoS One. 2023 Sep 8;18(9):e0290540. doi: 10.1371/journal.pone.0290540 (PMC10490972; doi:10.1371/journal.pone.0290540)
Supplement: S2 Appendix — (DOCX) [file pone.0290540.s002.docx]

Appendix II

Illustrative Quotes

**ATTITUDE CHANGES OVER TIME**

- *“I think I’m more convinced that I will not take it as time goes on.”*
- *“Mine hasn’t changed either. I just wish everybody would get theirs and maybe this pandemic would die down a lot.” -Vaccine favorable Veteran*
- *“I don't think I really had an opinion change on vaccines. I've always kind of felt when--I don't know if we're talking about just the COVID vaccine or vaccines in general, but I've just always grown up getting vaccines and feeling vaccinated was an important thing to do. I can't say that my opinion of vaccines has really changed in COVID. I definitely think that there can be some risks associated with them, but I'm willing to take those risks, I guess. And I'll just also add just with the Johnson & Johnson vaccine, my sister's family is a military family and they had to move overseas in COVID. And just seeing kind of the effectiveness of vaccines there, maybe it just reinforced my belief that vaccines work because on the military base, the military base in Japan is opening because the majority of the American citizens there on the military base are vaccinated, but a lot of people--most of Japan is not vaccinated, and COVID is very rampant there. So if anything, I know at the beginning I said maybe I haven't really been impacted, but maybe I believe even more that vaccines can have an impact.”*
- *“…[A]s long as all this is out there actually FDA approved and they’re not trying to slip some COVID stuff in there…I’d be willing to take it, again if it’s FDA approved… for a specific illness then yes…[b]ut I’m very cautious about it with everything that they’ve done this year making it, just throwing it out there and making it political.” -vaccine hesitant participant*
- *“For me, I've heard of some people who's had good experiences and some who've had not-so-good experiences and have had bad side effects, but for me I'm still on the nay for the vaccine. Don't want to do it just because I also feel like it's just was something that was rushed too soon and there's not enough evidence. For me personally, I feel like I've done my research and everything and I feel like it's just not enough and I don't understand what the push is on them pushing everyone to get the COVID vaccine and how all of a sudden, everyone has COVID but the flu has miraculously disappeared from the earth, and no-one gets the flu anymore. All of a sudden everyone gets COVID, so that's just my take on it.”*
- *“I think my attitude has stayed the same. At the beginning, I wasn't comfortable in getting it, but I decided to get it just because I work with the public in my work, studying, and then I also was going to be traveling with the elderly. And so I was getting it--that was my decision to get it, just because of that. But then once I had that side effect, then that just confirmed that I should have stuck to my heart and not get it. So, yeah. So I think it hasn't changed. So I ended up doing it but then I ended up going back.”*
- *“I don't necessarily think my attitude has changed. From the very beginning, when vaccines had first come out, it was kind of a little bit of an excitement that we were getting closer, and then when people started using them and nobody grew a third eye, and the zombie apocalypse didn't show up, we began to feel more and more confident in the science that these vaccines were safe and were working.” -vaccine-favorable participant*
- *“I guess honestly it’s just, I’m not real sure about the whole thing with my attitude over time of everyone’s been pushing it so much that it makes it almost worrisome to me. I’ve gotten myself, I mean obviously it’s a military forced vaccines or vaccinated but I would’ve anyway. My kids have always been vaccinated, no problems. So it just when it almost seems like they’re forcing it so much that it makes you, it makes me worried to get it. And that’s the only reason that I haven’t at this point because of that. I have not, no problem getting it soon in the future things like that but because it’s being pushed so much it almost seems like, to me there needs to be more research on it first.”*
- *“I was hesitant at first mainly because of misinformation from my family. But I think I was more just willing to get it after it was being offered to us but also because I interact with a lot of people that are in the medical field. And so I was able to get some information from them too. So I think that I was more willing to get it after that.”*
- *“[My attitude] changed for the better, I think, because after it seemed like the number of cases of deaths started to go down once the vaccine started rolling out, and that gave me more confidence that it was effective. I think I was a little bit nervous at first, but then once I saw what seemed to be the effectiveness of it, I think it gave me more confidence in going and doing it.”*
- *“I was actually, honestly, pretty convinced I was not going to receive the vaccine. And then after doing a bit of research and kind of just looking over some of the documentation on it, I felt like it was safe enough to go ahead and go forth with it. And then combine that with my job as well as having a high-risk person at home, it just made sense to go ahead and get vaccinated.”*
- *“I did feel a little bit nervous when there was some cases that reported blood clotting when some people had taken the vaccine. But even though it made me a little bit nervous, I still believe it was just a more rare case and that still getting the vaccine overall would be safer for me.”*

**BENEFITS OF GETTING THE VACCINE**

- *“I would think complete immunity from being, death if you will or being hospitalized under a ventilator. I wouldn’t wanna put my family nor myself through that. And the benefactor of being able to have some kind of shield of protection from a variant. Again with the Delta and then the other variant. Whether the efficacy goes down a couple percentages or so it still gives you some kind of protection. And so that’s my benefit from it.”*
- *“I can go back out again instead of being locked inside a house… I was just happy to be able to get out and not being worried of that if I get the COVID it’s gonna kill me. That was my main concern. I can finally get outta the house.”*
- *“I think a lot of people become a lot more relaxed after they got the vaccine. They tell me so. I mean it’s like, I think it helps with the anxiety. I mean I don’t have that issues because it’s not just vaccine that we have to do we have to do the other, you know COVID protection stuff. But I see a lot of people who just after the second dose they feel a little freer. And they definitely do feel freer. Just moving around or even getting together with relatives finally. Some people got it really for that reason ‘cuz they really wanted to see their grandkids or go visit all the relatives, so. I would say that’s a huge benefit for not just me but society.”*
- *“Some of the benefits of the COVID vaccine is, I feel more at ease going home. I live in Salt Lake, and I do interact with a lot more people than 30, which is my hometown. And in my house, we take care of my grandma, who's like 98 years old, so she's really high-risk. And then my other grandma also lives in the same place, and there's a lot of people who are just older who have weakened immune systems. And so, for me, I feel more comfortable going home because I know I've taken appropriate measures to keep them as safe as possible.”*
- *“The benefits would just be traveling and just kind of getting back to normal as fast as possible, or as soon as possible, getting to see your grandparents and anyone that's in immediate danger of COVID. So it was nice and reassuring when the elderly got the vaccine and you could still hug people and just be around people. That was the good thing about it.”*
- *“I already feel like there's a sense of relief that having the vaccine gives you. I feel like the more and more people are getting vaccinated, the more and more people are feeling a little bit more comfortable about their daily activities. Still being smart about their daily activities, but there's a sense of a kind of relief feeling, that there's a light at the end of the tunnel, so to speak, and that vaccine is what's going to kind of get us at least somewhat there.”*
- *One participant, self-described as vaccine-unsure believed the vaccine would most likely be helpful. They said, “I probably think that it'll help. Probably helps people not get it, or at least not die from it. I think I'd probably believe that. Maybe, maybe not. I think that's probably a good thing, in general.”*

**RISKS OF GETTING THE VACCINE**

1. GETTING SICK BC OF VACCINE

- *“I would also say, for me, it would just be the short-term risks of--I just had heard some people getting quite sick for that day. And so, I have three little kids. I've got twin two-year-old children and a newborn and so just the thought of being sick for one day was a big deal for me. So that kind of made me a little hesitant because I just can't afford to be sick. It's just hard to be a mom and be sick. But obviously, I wanted to get the vaccine and I wanted to be vaccinated, so anyway, that was just my short-term risk.”*

1. VACCINE SIDE EFFECTS

- *“Personal risks, Ball’s palsy, there’s been a documented list of heart issues, breathing problems, and of course there’s a ton of lawsuits out there right now that the government’s trying to push away for every single one of these for unknown reasons.”*
- *“This is going to feed into one of the conspiracy theories, but I think that there was a very low personal risk that was not adequately communicated because the vaccines have not finished phase three clinical trials, I believe, and I have not looked this up. I believe that phase one and phase two were completed, but the long-term large number of phase three clinical trials have not been finished. There was an emergency approval for use of the vaccines, and I understand that, and I have no issues with it, and I got in line just as soon as I could to get the vaccine, but I thought that there was a very low risk of long-term side effects that are not clearly understood still.”*
- *“Worry about the side effects or how the effects of the shot, I guess I was feeling that you know if whatever reaction I have, I’ll have it. But I’m very physically strong internally so you know I think after the second injection I got maybe a 18, 20 hours of body aches and that’s sometimes what I get from the flu shot. So there was no, no difference for me.”*
- *“The only one [risk] I knew about was getting flu-like symptoms. That was the only real risk that I knew of. And the workers that I had underneath me, one got deathly ill. Not really that ill, but got very, very sick. I was tired. I just was sleepy, which is something I deal with every day, so it wasn't that big of a change for me.”*

1. INFERTILITY

- *“I'm concerned about long-term risk with fertility and, even in the moment, breastfeeding and how components of the vaccine may pass through to my infant. I guess another risk is heavy metal content and heavy metal detoxification in my body post vaccine. For me, the side effects of COVID for people in my age group seem a lot better than the vaccine side effects for people, and those are the risks that I made my decision based on.”*
- *“Like I mentioned, just the whole infertility thing is really what's on my mind, because it's something that I'm currently facing, so. And I've done lots of research. I've talked to several nurses, my doctors, and to me, I just don't feel like that research is there, and what is there, has kind of been on my side. So I just feel like definitely the more research with different things like that.”*

1. *NOT ENOUGH “PROOF” OF EFFECTIVENESS*

- *“When I first went to get the vaccine of course in my mind I thought, you know, this it really hasn’t had a lot of proof behind it, and like with any shot you take, there’s always gonna be a risk. But I knew if I didn’t get it, there was a higher risk of becoming sick, or sicker. So I went and got the shot, and I’ve been happy I did it. And it within I guess a week after I got the shot, those fears that I had at the beginning were laid to rest. And I think when you go to get something new there’s always gonna be apprehension. And then if you come out of it okay, cool. It’s like, you know, taking off the training wheels off the bike, am I gonna make it or not?”*

1. UNKNOWN SIDE EFFECTS

- *“I'm a pretty healthy person and I don't have any underlying conditions. And so I just feel like, if I'm doing that, and then how I got sick and the side effects or the blood clot. I'm just feeling like why risk it, to have those type of effects, when I'm not sick already, you know what I mean? And so I think that's what I'm scared of, is the long term, not knowing what will happen.”*
- *“For me, the biggest [risk] was—well, and this is new. It's myocarditis when your heart muscles actually swell up. That was associated with the mRNA vaccines, which is what I got. Aside from that, it's just the fear of the unknown. These are new. They were expedited. And so the long-term effects, nobody really can tell you.”*

**SOURCES OF INFORMATION**

1. SELECTED DOCTORS, RESEARCHERS OR ANECDOTES

- *“Well definitely don’t trust the news. People, I’ve got some friends that are doctors and they give me some accurate information. And even they tell me not to get the vaccines. And of course with a special needs child I talk to their doctors constant, his doctors constantly. And they give me some accurate stuff about what they’re seeing out there.”*
- *“I try to stay abreast of trends and look at some of the Journals of Medicine, but I probably put my heaviest weight on just some of the anecdotal stories, although that’s not scientific, from people that I know that are hospital managers or nurses. And when I found out that one of the local hospitals here that they’re less than 50% of the hospital staff had opted to take the virus so far, excuse me, the vaccine for the virus so far that made me kind of think well, you know, if healthcare workers have such low rate of wanting to take the vaccine then maybe it’s not as bad as some people say. And every time I’ve talked to friends that are nurses and doctors or hospital administrators, the ones that they tell me that have died, that they can talk about, from COVID, they’ve all had some real bad pre-existing health conditions and/or they were eventually ended up on a ventilator. And I guess these folks could have been killed if they would have gotten pneumonia or the flu. You know, extreme elderly or folks that have advanced diabetes or heart disease and some of these other things.” -Vaccine-hesitant participant*
- *“Yeah it would have to be a very select few scientist and doctors and well-educated researchers. Just ‘cuz somebody has a PhD behind their name doesn’t mean I’m gonna trust ‘em.” -Vaccine-hesitant Veteran*

1. TRUSTED SOURCES INCLUDE GOV’T ORGANIZATIONS, OFFICIALS, AND NEWS OUTLETS

- *“I think from the standpoint of if you’re gonna wait for the media to give you the information they’re kinda wishy-washy. And it’s kinda hard you’ve almost gotta interpret what they’re trying to say. And I’m not, my problem with the media is that they, they kinda, they don’t take one side or the other they kinda like go down the middle. And not give people a real clear understanding of what’s going on. The best information I get is from the VA. I get it through my email and they’re sending updates and something on my iPad and my phone updating you on what’s happening, what’s available, how it’s affecting people, and what they’re doing to make things better. So I think the best information I get is from the VA.”*
- *“I like to get mine from Dr. Fauci. I like to get mine from, what is she the like the director of CDC. There’s two medical consultants on the news reports, I like to listen to them. We here, we have all kinds of fliers on our communication site. There’s always updates from the Montana Public Health Association and Lake County. So I mean we’re just getting inundated with information. So if you want it, it’s there.”*
- *“You know, I kinda trust most of the sources that you see on tv. I mean everyone has their opinion, the CDC, you know, everyone, I kinda look for our local news to help us out, especially in our area, that they’ve been a trusted source of information since this whole COVID thing began. I get bothered when they start putting a political label to it. I kinda ignore that area. Because, you know, the disease doesn’t care who you are, or what you are, or how you vote, it’s gonna get you if you get it. So, what the news media, if you’re careful which one you listen to, you’re gonna get some straight facts. You know, there are some big ones out there that lean one way, and other one leans another way, so you just gotta be careful. And there’s no better information than going to find out yourself. You know the old saying, don’t believe anything you read and half of what you see, or something like that.”*
- *“For me the CDC because all the health department gets the information from the CDC. And the doctors get it from the medical, health department or the CDC. So it trickles down. -Vaccine-favorable Veteran*
- *“I trust certain news sources, local news, I trust information coming from the CDC, I trust the information coming, yes, the information coming from the CDC, doctors that are appointed in the, to deal directly with this virus. So, information from people like that. Sources like that.”*

1. UNTRUSTED SOURCES INCLUDE MEDIA, POLITCIANS, SOCIAL MEDIA, CDC, GOV’T ORGANIZATIONS

- *“I didn’t really go to the CDC they kept changing their mind every other month. So I didn’t even bother going through them. I went more through the universities and the medical research facilities. When the doctors are saying one thing and then two or three days later another set of doctors come out, contradict the doctors had said before them, after about that yo-yo about four or five times the CDC, I just ignored them.” -female Veteran*
- *“If you’ve got any strongly politically influenced news media outlet I’m always gonna be suspect. It doesn’t matter if I’m for you know, against, whatever, whatever side it is. I’m always gonna be suspect because they have, there’s other motives as to why they’re pushing one way or the other. So for me it’s, you know it’s, it would be more of an entertainment if I am gonna watch either side. Just to see, for me it’s just more entertainment. I’m not gonna take stock in what they’re pushing.”*

1. MIX OF TRUSTWORTHY AND UNTRUSTWORTHY SOURCES; REC’D INFO ON HOW AND WHERE TO GET VACCINE

- *“I got a, locally here I’ve gotten little bitty cards kinda like a postcard said vaccines are available from this date to this date here at this location, from this date to this date at this location, and so forth.”*
- *“Started to see it I guess back in January, February, when all this started go down, information of how to get your shot, what websites to go look for, how you can get it, the VA was sending out emails, and texts, and things like that, and local tv stations were telling us what to do, they were giving us numbers of how many people were coming down with it, and all the deaths. Now we’re still getting information on the tv about . . . people are vaccinated and the percentages what, the information as to where to get your shot, but that dwindled almost to nothing around here. You know, I think they rely on word of mouth sometimes where to go get it. But like your local pharmacies, the big chains still offer it, and you can still get it, but it’s not advertised unless you see the sign out front of the, you know, one gentleman said the grocery store, you know, the pharmacies and stuff, it’s kinda slimmed down. Now they’re just telling you on the news about who continues to pass and what’s the percentage of people that got vaccinated. So it’s at first you’re overloaded with it, and now it’s hardly anything.”*

1. NOT ENOUGH AVAILABLE INFO, DIFFICULT TO UNDERSTAND, CONTRADICTORY

- *“I don’t think there’s a lot of information out there”. -Vaccine-hesitant Veteran*
- *“And I think even if you are fairly well versed in reading peer reviewed journals there’s a lot of information that is confusing or conflictory, especially for a lay person who’s not in the medical field but you can talk to medical people who knows how to read surveys. It’s kind of hard to dial down on what the truth is but what I have gathered is for most folks who are in reasonable health this is not terribly dangerous.” -Vaccine-hesitant Veteran*
- *“I think it’s been pretty accessible for anybody who’s willing to look for it. To put in the work to like actually find updates and information on it. As far as credible information goes like it’s there if you look for it. I don’t think it’s been hidden just maybe kind of buried under tons of other much more easily accessed misinformation and so on.” -Vaccine-favorable Veteran*
- *“I don’t think there’s like any more information that I would like to get. I mean as far as you know what they’re already saying. I would just like to get updates on how the vaccine is handling these variants probably. And that would probably be it. And if anymore variants pop up and how the vaccine is working against those.” -Vaccine-favorable Veteran*

1. DID NOT RECEIVE MUCH INFO, NOT TRANSPARENT, SKEWED POSITIVE

- *“I don't remember receiving anything directly. I mean, it was just indirect information from other sources. Nothing that was targeted, that I recall.” -Rural community member*
- *“The only information I got was what I went out and looked up. Like I said before, I think too much of the information is being held back. It's just, "Get it, get it, get it, get it. Do it or else. Get it, get it." Everybody's just saying get it. They're not saying why, they're not saying how it's made. You got to go up and look that up yourself and if you do there's so many conflicting things. So any information I got was only by myself. And so I think the transparency's just not where it needs to be.” -Educator*
- *“Other than where to get the vaccine, I feel like we're given very little information other than locally. I mean, the news reports and CDC reports and you got to follow these sites to get information. But as far as any community campaigning, I don't think there's a lot of information other than the vaccine will protect you, the length of time it takes to get between vaccines and the two weeks after getting the second dose, and then more about lifting restrictions. But I don't really feel like there's a whole lot of information out there other than if you seek it out. It's just not readily there for us to understand.” -LTCF staff member*
- *“To me, I feel the only--it's nothing but positives. You don't hear anything negative about this vaccine at all. And they're not reporting any dangers or side effects. And I just feel it's not being communicated 100% to what the bad parts of it. I feel like it's all just celebrities pushing it and, "Get vaccinated so you can hug your family." And I feel like that's all we see. But as far as the medical side of it, I really know nothing. You can't find a lot of information without it being like, "Oh, this is fake news," or, "Fact-checkers haven't—this isn't real. So I don't know what to believe at this point.” -Underrepresented community member*

1. **SCARCE INFORMATION; DIFFICULTY FINDING ACCURATE AND ACCESSIBLE INFORMATION**

- *“I found early on from YouTube and MedCram, and I listen to a lot of their virologists and scientists. And I got a lot of information from MedCram. And I didn't trust any other information that I got.” -Rural community member*
- *“I go to the CDC when I need to find out about my work setting and what I need to do to protect my certain business. I thought it was interesting that my neighborhood gathered together to do a vaccination for anyone 12 and above at our church parking lot. And that was just cycled through emails, and I thought that was great.”-Underrepresented community member*
- *“Well, this might not be a popular answer, but I'm just going to say Dr. Fauci. I love the guy. He's a great presence on television, and he was able to sort of keep his jobs through the whole end of the Trump era. But I don't know, you know what? I mean, he's kind of the face of the coronavirus in the United States. And he is a trustworthy guy. I like to listen to him. I like to hear what he says. And he seems to be kind of commonsense and not too radical one way or the other.” -Educator*
- *“For me, personal doctor, though I haven't talked to him about it, but I have a lot of relatives who are doctors. The last people I trust is the media and government officials. I would trust them a lot more if they weren't pushing the vaccine for people who do not need it like small children, two years of age, and those who've recovered from the virus. They do not need the vaccine. There's very little upside, benefit for them and the risk, and yet they push it. And they lose credibility, for me.” -Conservative community member*
- *“Maybe this is opening a can of worms, but I feel like the AAP has been very trustworthy. I feel like the CDC has demonstrated in the past few years that it can be, at times, a political tool and will, at times, give out bad information if pressured. So interestingly, my trust in the CDC has been pretty damaged over the course of the past couple years. But I do feel like the AAP has stood firm and stood by kind of what the data supports.” -Primary care physician*
- *“It's been disappointing to see how they have politicized this urgent public health situation. It's made it difficult because ordinarily, you can read professional peer-reviewed journals, and there's been even false things published in there, and then they have to retract them. One of the problems is that they make a big deal about it when it's published. But when it comes out later that it was false or distorted or what have you, they don't make a big publication about, "Oh, that was misinformation." That's not helpful. Also, people like Fauci who keeps changing his mind back and forth and back and forth, one of the things that's been--that just makes it hard. If the CDC can't be a good source for you to feel confident, it causes unnecessary fear if you can't trust what's in the professional journals that the doctors and researchers are writing, that's unnecessary fear. Because if I wanted to be able to have a discussion with my doctor and you take the article in and then six months later, or now it's like a year later we're finding out where there's been things hidden, the truth is coming out, that's very discouraging and concerning because you want to be able to trust these professionals.” -Female community member*
- *“I guess I would trust the anecdotal evidence over course of time because the medical community, sometimes they change things. I'm not a clinician or anything, but that's just what I observe, so that's why I'm a little--I'm a paranoid person, so I have to see how it runs its course. But yeah, I guess WHO, I could trust them a little bit.” -Underrepresented community member*
- *“I was just going to say that, personally, with something that has become so politically charged, I don't feel like I can trust any media outlet at this point. There are definitely some that I feel more inclined to trust, but I just feel that because, at the end of the day, they're just trying to make money and have headlines, I don't like that motive, and so I need to look for other sources that are actually about science and not just about people trying to get as many clicks on their article as they can or as many views on their show because it just seems like there's a lot of room for overexaggeration or perhaps underexaggeration and misinformation.” -Young adult community member*
- *“Nowadays, it's just getting even harder to trust things. I do trust the CDC. I do trust the government. But I also see how the government has been so into managing this when actually, it should be other professionals who are actually on the field, who should be giving us the information, managing all of this, instead of the people in government who don't know anything about it. So, yeah. I don't trust social media either. It's not safe, what you find there.” -Underrepresented community member*
